# Ligation of Glycophorin A Generates Reactive Oxygen Species Leading to Decreased Red Blood Cell Function

**DOI:** 10.1371/journal.pone.0141206

**Published:** 2016-01-19

**Authors:** Joseph Khoory, Jessica Estanislau, Abdallah Elkhal, Asmae Lazaar, Mark I. Melhorn, Abigail Brodsky, Ben Illigens, Itaru Hamachi, Yasutaka Kurishita, Alexander R. Ivanov, Sergey Shevkoplyas, Nathan I. Shapiro, Ionita C. Ghiran

**Affiliations:** 1 Division of Allergy and Inflammation, Beth Israel Deaconess Medical Center and Harvard Medical School, Boston, United States of America; 2 Department of Surgery, Brigham and Women’s Hospital and Harvard Medical School, Boston, United States of America; 3 Department of Neurology, Beth Israel Deaconess Medical Center and Harvard Medical School, Boston, United States of America; 4 Department of Synthetic Chemistry & Biological Chemistry, Graduate School of Engineering, Kyoto University, Katsura, Kyoto, Japan; 5 The Barnett Institute of Chemical and Biological Analysis, Northeastern University, Boston, United States of America; 6 Department of Biomedical Engineering, Cullen College of Engineering, University of Houston, Houston, United States of America; 7 Department of Emergency Medicine and Center for Vascular Biology, Beth Israel Deaconess Medical Center and Harvard Medical School, Boston, United States of America; Massachusetts Institute Of Technology, UNITED STATES

## Abstract

Acute, inflammatory conditions associated with dysregulated complement activation are characterized by significant increases in blood concentration of reactive oxygen species (ROS) and ATP. The mechanisms by which these molecules arise are not fully understood. In this study, using luminometric- and fluorescence-based methods, we show that ligation of glycophorin A (GPA) on human red blood cells (RBCs) results in a 2.1-fold, NADPH-oxidase-dependent increase in intracellular ROS that, in turn, trigger multiple downstream cascades leading to caspase-3 activation, ATP release, and increased band 3 phosphorylation. Functionally, using 2D microchannels to assess membrane deformability, GPS-ligated RBCs travel 33% slower than control RBCs, and lipid mobility was hindered by 10% using fluorescence recovery after photobleaching (FRAP). These outcomes were preventable by pretreating RBCs with cell-permeable ROS scavenger glutathione monoethyl ester (GSH-ME). Our results obtained *in vitro* using anti-GPA antibodies were validated using complement-altered RBCs isolated from control and septic patients. Our results suggest that during inflammatory conditions, circulating RBCs significantly contribute to capillary flow dysfunctions, and constitute an important but overlooked source of intravascular ROS and ATP, both critical mediators responsible for endothelial cell activation, microcirculation impairment, platelet activation, as well as long-term dysregulated adaptive and innate immune responses.

## Introduction

In humans, during normal conditions, low concentrations of complement-opsonized circulating inflammatory particles are bound to red blood cells (RBCs) via complement receptor 1 (CR1) and delivered to resident sinusoidal macrophages in the liver and spleen, a process known as immune-adherence clearance [1,2]. During periods of excessive complement activation, nascent C3b and C4b fragments bind irreversibly through thioester bonds to hydroxyl groups of heavily glycosylated glycophorin A (GPA) on circulating RBCs [3]. Our results using RBCs isolated from SLE [4] and trauma patients demonstrate that binding nascent complement fragments C3b and C4b to human RBC plasma membranes has significant negative effects on RBC function, namely β-spectrin phosphorylation and decreased membrane deformability [5,6]. *In vitro* engagement of GPA by complement fragments significantly impacts membrane protein functionality, leading to decreased diffusion of key complement regulatory proteins, namely CD35, CD55 and CD59, and promoting the formation of new protein complexes between GPA/band 3/ankyrin and spectrin that directly impacts membrane deformability [7]. Furthermore, GPA ligation has been associated with increasing RBC membrane rigidity, which impairs RBC flow through the microcirculation [8]. The mechanical and biochemical consequences on human RBCs following GPA engagement either by naturally occurring auto-antibodies [9] or complement fragments [5–7] may represent an initial signal triggering a cascade of events culminating with the premature removal of altered RBCs from circulation. Acute inflammatory conditions associated with complement activation have been correlated with reductions in capillary flow and corresponding decreases in microcirculatory perfusion [10,11].

Reactive oxygen species (ROS) are constantly produced by RBCs through oxygen transport, generating high levels of oxidative stress that significantly reduces its lifespan [12]. Mature RBCs lacking biosynthesis machinery rely exclusively on enzymes produced earlier in development to maintain ROS scavenger systems. Genetic or acquired conditions associated with decreased ROS scavenging capabilities can cause extensive membrane damage leading to reduced RBC half-life and intravascular hemolysis [13]. Acute and chronic inflammatory conditions associated with complement activation promote a sustained increase in total blood ROS content that parallels disease severity. Whether RBCs could be a source of intravascular ROS during acute and chronic inflammatory conditions is currently unknown.

In the past decade, an intravascular purinergic overload critical in initiating, maintaining, and propagating host maladaptive inflammatory responses has begun to be revealed in diseases associated with excessive complement activation [14–17]. A detrimental effect of ATP and adenosine receptors (P2Y2 and A3) in the host’s response to injury using a murine model of septic shock was also reported. Mice lacking either P2Y2 or A3 receptors showed reduced organ damage and mortality following septic shock [18]. These data strongly argue that ATP and its by-products, acting on their cognate receptors, are essential for the disproportionate PMN and T cell inflammatory host responses that characterize acute and chronic inflammatory conditions such as sepsis. The source(s) of increased circulating ATP is currently not known, and the molecular mechanisms regulating ATP release remain poorly defined.

Our results show that GPA ligation induces a NADPH oxidase-dependent increase in the intracellular concentration of ROS, which, in turn, trigger multiple downstream cascades leading to caspase-3 activation, increased band 3 phosphorylation, and significant RBC ATP release. Functionally, excessive ROS production has a direct, detrimental effect on RBC membrane deformability and lipid mobility, outcomes that are preventable or partially reversed by incubating RBCs with cell permeable ROS scavengers. Our results obtained *in vitro* using anti-GPA antibodies were partially validated using RBCs isolated from septic patients, suggesting that during inflammatory conditions, circulating RBCs could constitute an important but overlooked source of intravascular ROS and ATP, both critical mediators responsible for endothelial cell activation, and microcirculation impairment.

## Experimental Procedures

### Antibodies

Antibodies were obtained as follows—LEAF purified mouse IgG_1_ (401404); LEAF purified mouse IgM (401604, Biolegend, San Diego, CA, USA); mouse anti-phosphotyrosine mAb (Life Technologies, Carlsbad, CA, USA); mouse monoclonal anti-beta-actin Ab (A2228), mouse monoclonal anti-glycophorin A IgG (clone R10, Lot B0607, sc-53905); mouse monoclonal anti-GPA IgM (clone E4, G7900, Sigma-Aldrich, St Louis, MO, USA); anti-CR1 mAb 1F11 (gift of Henry Marsh, Celldex Therapeutics, Needham, MA, USA); mouse monoclonal anti-CFTR (CF3) (ab2784, both Abcam, Cambridge, MA, USA); AffiniPure goat anti-mouse IgG (115-005-003); ChromPure rabbit IgG (011-000-003); ChromPure mouse IgG (015-000-003, Jackson ImmunoResearch, West Grove, PA, USA); HRP conjugated goat anti-mouse antibody (Millipore, Billerica, MA, USA); mouse monoclonal anti-human C3d (A207), mouse monoclonal anti-human C4d (A213, Quidel, San Diego, CA, USA).

### Reagents were obtained as follows

2–2 Zn(II) ATP Probe (gift from Dr. Itaru Hamachi, Kyoto, Japan); Vybrant® FAM Caspase-3 and -7 assay kit (V35118), Eosin-5-maleimide (E118), 1,2,3-dihydrorhodamine (D24806), Hank’s balanced salt solution with Ca^2+^ and Mg^2+^ (HBSS^++^) (14025) or without Ca^2+^ and Mg^2+^ (HBSS^—^) (14175), YO-PRO^®^-1 iodide (λ ex/em 491/509) (Y3603), NuPAGE LDS Sample Buffer (NP0007, Life Technologies, Carlsbad, CA, USA); IgG and protease-free BSA (Jackson ImmunoResearch, West Grove, PA, USA); ATP (A2383-5g), L-glutathione, reduced (G4251), Luperox^®^ TBH70X tert-butyl hydroperoxide solution (458139), Diphenyleneiodonium chloride (DPI, D2926), sodium orthovanadate (S6508), MK-571 sodium salt hydrate (M7571), ovalbumin (A5503, Sigma-Aldrich); ENLITEN^®^ ATP Assay System (FF2021), rATP (P1138, Promega, Madison, WI, USA); NSC23788 (553502), glutathione monoethyl ester (353905), GlyH-101 (219671, EMD Millipore, Billerica, MA, USA); 8-bromo cAMP sodium salt (1140), calphostin C (1626), indomethacin (1708), iso-PPADS (0683), KT5720 (1288), Phorbol 12-myristate 13-acetate (PMA) (1201), PPADS (0625), Procaspase-activating compound (PAC) 1 (2581), suramin hexasodium salt (1472), Z-DEVD-FMK (2166, R&D Systems, Minneapolis, MN, USA).

### Preparation of Red Blood Cells (RBCs)

Blood was obtained from healthy adult volunteers in accordance with the guidelines of, and approved by the Institutional Review Board of Beth Israel Deaconess Medical Center. Donors from multiple diversities were recruited by local advertising and written, informed consent was obtained from each volunteer in accordance with the Declaration of Helsinki. Approved study staff were responsible for all blood collection, processing, and documentation of consent files. Red blood cells were isolated as previously described [19]. For flow cytometry and microscopy experiments, 20–80 μL RBCs were harvested by finger prick and collected into a microcentrifuge tube containing HBSS^++^ with 0.05% IgG-free BSA. RBCs were centrifuged (5,000 x g, RT, 1 min), and supernatant was removed by aspiration. This step was repeated 2 times.

Criteria for Sepsis and Septic Shock Patient Samples–For this study, "sepsis" was defined as meeting systemic inflammatory response criteria in the setting of infection, whereas severe sepsis was defined as sepsis plus organ failure. These definitions are based on the American College of Chest Physicians/Society of Critical Care Medicine Consensus Conference [20].

### ATP release measurement from RBC

RBCs (2% Hct in HBSS^++^, 0.05% BSA) were placed in 1.5 mL reaction tubes and mixed with inhibitors or activators already placed in the tubes to minimize stimulation. Following incubation (RT x 10 min), RBCs were centrifuged (500 x g, RT, 2 min), the supernatant was discarded and RBCs were resuspended in buffer using an electronic pipette with the appropriate inhibitor or activator or buffer alone and again centrifuged (500 x g, RT, 2 min). Five replicate aliquots were placed into black, flat-bottom, 96-well plates, and ATP concentration was measured as described before [19].

### Hemolysis

Remaining cell suspensions were centrifuged (5,000 x g, RT, 2 min) and 80 μL of supernatant was measured at 415 nm using a VersaMax spectrophotometer (Molecular Devices, Sunnyvale, CA, USA). Experiments were excluded from this study if RBC hemolysis exceeded 0.25%

### Immunoblotting

RBCs were analyzed as previously described [21]. Nitrocellulose membranes were developed with SuperSignal^®^ West Pico or Femto Chemiluminescent Substrate [22] and imaged using the LAS 4000 imaging system (Fujifilm).

### Flow Cytometry

RBCs were analyzed using either FACScan or LSR II (BD Biosciences, San Jose, CA, USA). In all experiments ≥ 100,000 events were recorded and analyzed using FlowJo software Version 9.3.3. For each figure, a representative histogram displaying the mean fluorescent intensity [23] is shown, in addition to a cumulative graph for all experiments conducted.

### Measurement of complement components

Complement deposition on human RBCs was measured by incubation (37°C, 30 min) of RBC (0.5% Hct) with either mAb C4d, mAb C3d, or mAb IgG control (1:100) as described before [24]

### Measurement of ROS, activated caspase-3 and phosphorylated band 3/AE1

RBC (1% Hct in HBSS^++^, without BSA) were incubated with 5μM dihydrorhodamine (DHR) 123 (37°C x 30 min). Activated caspase-3 was detected with Vybrant^®^ FAM Caspase-3 and -7 Assay Kit. Phosphorylated band 3 was determined using eosin-5-maleimide (EMA, 37°C x 30 min).

### Fluorescence Recovery After Photobleaching

RBC lipid mobility was measured by incubating (RT x 30 min) RBC (10% Hct in HBSS^++^) with DiO lipid dye (15 μg/mL) and washed prior to addition of antibodies or reagents. RBCs were imaged using a 60x objective on an Olympus microscope and bleached with Vector Controller Laser and analyzed as described before [19].

### RBC Membrane Deformability

2D microchannel arrays were used as described in [25]. The cells were recorded using a 40 x 0.75 Ph2 Plan Fluorite objective on a TE300 Nikon-inverted microscope (Nikon Inc., Melville, NY, USA), using a Retiga EXi (QImaging, Surrey, Canada) CCD camera controlled by iVision 4.7 software (BioVision) at a rate of 10 frames/s. Movies were analyzed off-line frame by frame, and RBCs that displayed unusual shapes, overlapped, or clustered were excluded from measurement. For each experimental condition at least 50 RBCs were counted.

### Statistical Analysis

All flow cytometry experiments were analyzed using FlowJo version 9.6.4 (TreeStar) or higher. Statistical tests were performed using Prism version 4.0 (GraphPad). All error bars represent the mean of 5 readings + S.D. unless noted otherwise.

## Results

### Ligation of GPA stimulates ROS production

**Fig 1:**
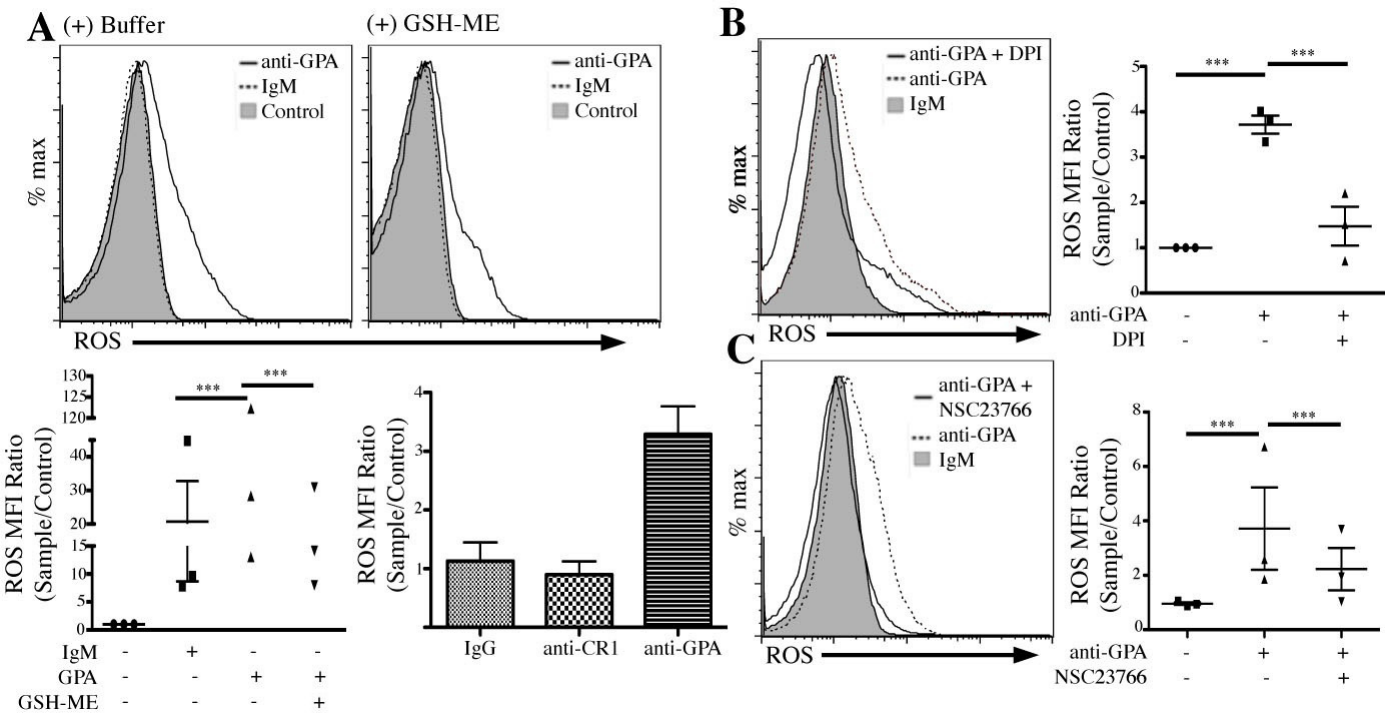
GPA ligation on human RBCs stimulates ROS production via Rac 1 and NOX. (A) Ligation of GPA promotes ROS production in RBCs; Cell permeable GSH-ME reduces ROS. RBCs preloaded with DHR 123 were treated with anti-GPA **(**mAb E4, 1 μg/mL) or IgM control mAb in the presence or absence of 1 mM GSH-ME (RT x 10 min). Cells were washed twice and recorded by flow cytometry. **Lower panels:** RBCs were incubated with either IgG or anti-CR1 under similar conditions (bottom right). (B) GPA-induced ROS production dependent on NOX. RBCs preloaded with DHR 123 were treated with either control IgM or anti-GPA (mAb E4) in the presence or absence of NOX inhibitor, DPI (10 μM, 37° x 60 min). RBCs were washed twice and analyzed by flow cytometry. Right panel shows cumulative results of the same experiment. (C) GPA-induced ROS production dependent on Rac 1. RBCs preloaded with DHR 123 were treated with either control IgM or anti-GPA (**mAb** E4) in the presence or absence of **Rac 1 inhibitor,** NSC23766 (500 μM, 37° x 60 min). RBCs were then washed and analyzed by flow cytometry.

DHR 123-loaded RBCs were incubated with tert-butyl hydroperoxide (t-BHP), a known ROS promoter. t-BHP (10, 100 μM) increased intra-RBC ROS production in a concentration dependent manner (**S1 Fig**), from MFI = 35.7 in control RBCs to 49.6 and 1164, respectively. For *in vitro* experiments, we elected to ligate GPA with monoclonal antibodies (mAb), which induce profound changes in human RBCs during pathologies associated with naturally occurring anti-GPA antibodies [9]. Ligation of GPA with mAb anti-GPA (**Fig 1A, top left panel**) increased intra-RBC ROS production over control, from MFI = 7.9 to 16.5, and pretreating RBCs with the cell permeable ROS scavenger GSH-ME (1 mM) reduced GPA-mediated ROS increase below basal levels (MFI = 6.18, top right panel), indicating that at basal conditions circulating RBCs contain low but measurable levels of ROS. To confirm that ROS production was due to the direct and specific engagement of GPA, we ligated RBC CR1, which we have shown increases RBC membrane deformability [25] and triggers a robust ATP response [19]. Here, ligation of RBC CR1 did not promote any measurable increase in intra-RBC ROS production (**Fig 1A, lower right**), suggesting that: a) GPA-mediated RBC ROS generation is not due to a non-specific response of RBCs to engagement of membrane proteins by antibody, and b) the increase in partially inhibited by the cell permeable ROS scavenger, GSH_ME (**Fig 1A, lower left panel**). Recently, NADPH oxidase [26] was identified as a critical enzyme in RBC ROS production, both in healthy and sickle RBCs [27]. We used DPI, a potent NOX inhibitor, to test if GPA-mediated ROS production is dependent on NOX activity. Inhibition of NOX activity by preincubation of RBCs with 10 μM DPI decreased the intracellular ROS concentrations in GPA-ligated RBCs from MFI = 35.4 to MFI = 19.5 (IgM control MFI = 12.2) (**Fig 1B**) suggesting a significant role of NOX in GPA-mediated ROS production. In addition, the GTP-ase protein Rac1 was recently implicated as an activator of NOX in human RBCs[27], and therefore critical to RBC ROS production. To test the involvement of Rac1 in GPA-mediated ROS production we next treated RBCs with a cell permeable Rac1 inhibitor (NSC23766) prior to GPA ligation, and observed a similar decrease in ROS levels, from MFI = 26.3 to MFI = 14.8 **(Fig 1C),** suggesting that GPA ligation induces an increase in RBC ROS production that is mediated by Rac1 and NOX.

### GPA ligation induces caspase-3 activation through increased intracellular ROS

**Fig 2:**
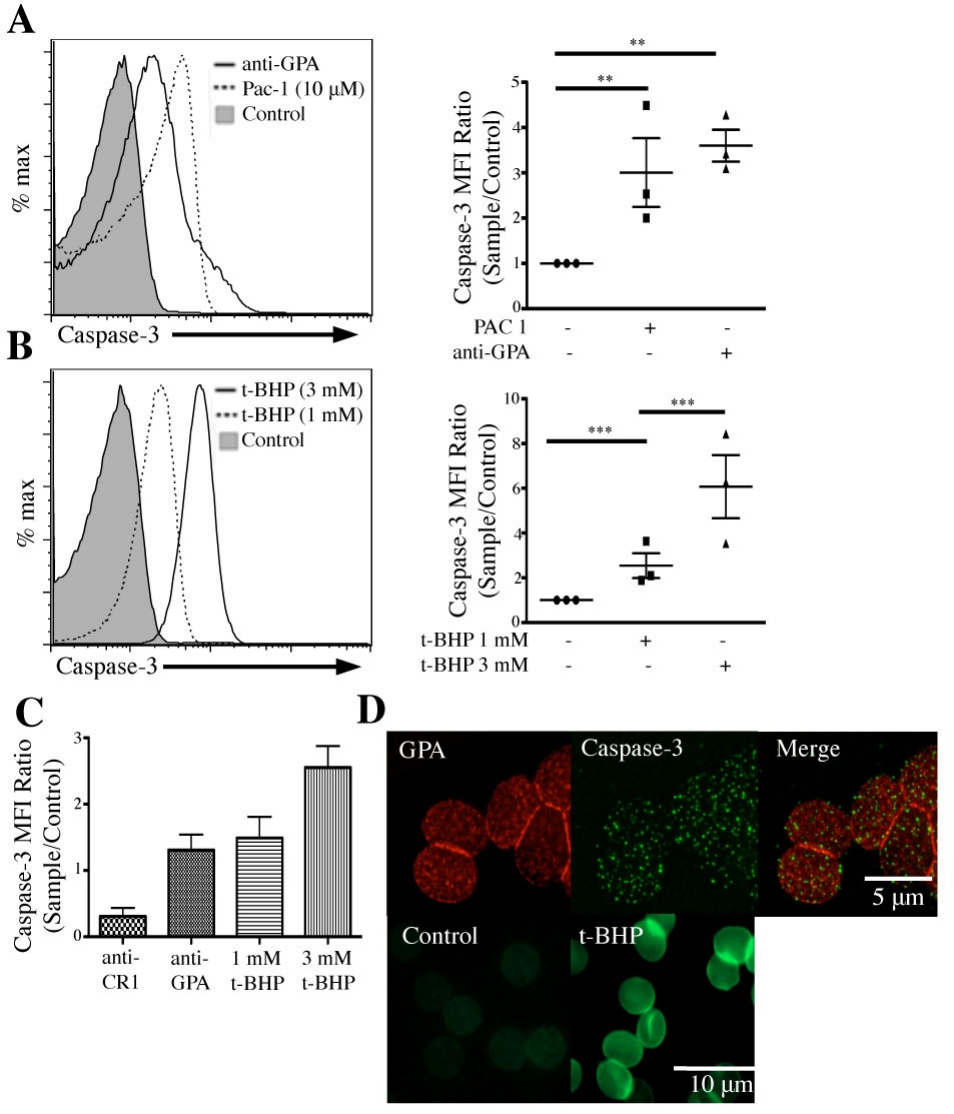
GPA ligation induces ROS dependent caspase-3 activation. (A) GPA ligation activates caspase-3. RBCs preloaded with FLICA (1X) were incubated **with** IgM control, PAC-1 (10 μM), or anti-GPA (**mAb** E4). Cells were washed twice and analyzed by flow cytometry. (B) ROS induces caspase-3 activation in RBCs. RBCs pretreated with FLICA were incubated with buffer, 1 mM or 3 mM t-BHP. RBCs were washed and analyzed by flow cytometry. (C) Cumulative data including RBCs incubated with IgG anti-CR1 as a negative control. (D) Microscopy images illustrating caspase-3 activation in human RBCs. RBCs pretreated with FLICA were incubated with anti-GPA mAb (E4, 2 μg/mL, top panels), buffer, or 10 μM t-BHP (bottom panels), followed by Alexa 594 fluorescent secondary antibody. RBCs were washed and loaded onto a microscope slide. Images of GPA ligation (left) and caspase-3 activation (center) were taken using an OlympusBX62 fluorescence microscope and further analyzed using 3D deconvolution on Slidebook Pro. The images were then merged together (right). The bottom images were taken using fluorescence microscopy.

In the past, others have shown a correlation between intracellular ROS accumulation and caspase-3 activation [28]. We therefore asked if GPA-induced ROS production activated caspase-3. RBCs pretreated with FLICA were incubated with either control IgM, anti-GPA (E4), or 10 μM PAC-1 (positive control) for 15 min at RT. Using flow cytometry, we observed a 4.3x increase in caspase-3 activation in GPA-ligated RBCs **(Fig 2A)** compared to IgM control, (MFI = 28.3 vs. MFI = 6.6), similar to our positive control (MFI = 23.1). Incubation of RBCs with known ROS promoter t-BHP generated a dose dependent increase in caspase-3 activation from basal MFI levels of 3.52 to 39.6 (1 mM) and 99.4 MFI (3 mM, **Fig 2B, left)**. Similar to our previous results **(Fig 1A)**, incubation of RBCs with anti-CR1 did not trigger activation of procaspase-3 **(Fig 2C)**, also suggesting that GPA-dependent activation of caspase-3 is not a non-specific response of RBCs to GPA ligation. We next investigated the pattern of caspase-3 activation induced by GPA ligation by fluorescence microscopy **(Fig 2D).** Interestingly, the distribution of activated caspase-3 showed a punctuated pattern near the membrane following GPA ligation **(top panels)**, and a uniform cytoplasmic staining following t-BHP-dependent activation (**bottom panels)**. Taken together, our data suggest that ligation of GPA triggers an increased intracellular production of ROS that is responsible for activation of procaspase-3.

### GPA-mediated ATP release depends critically on intracellular ROS

**Fig 3:**
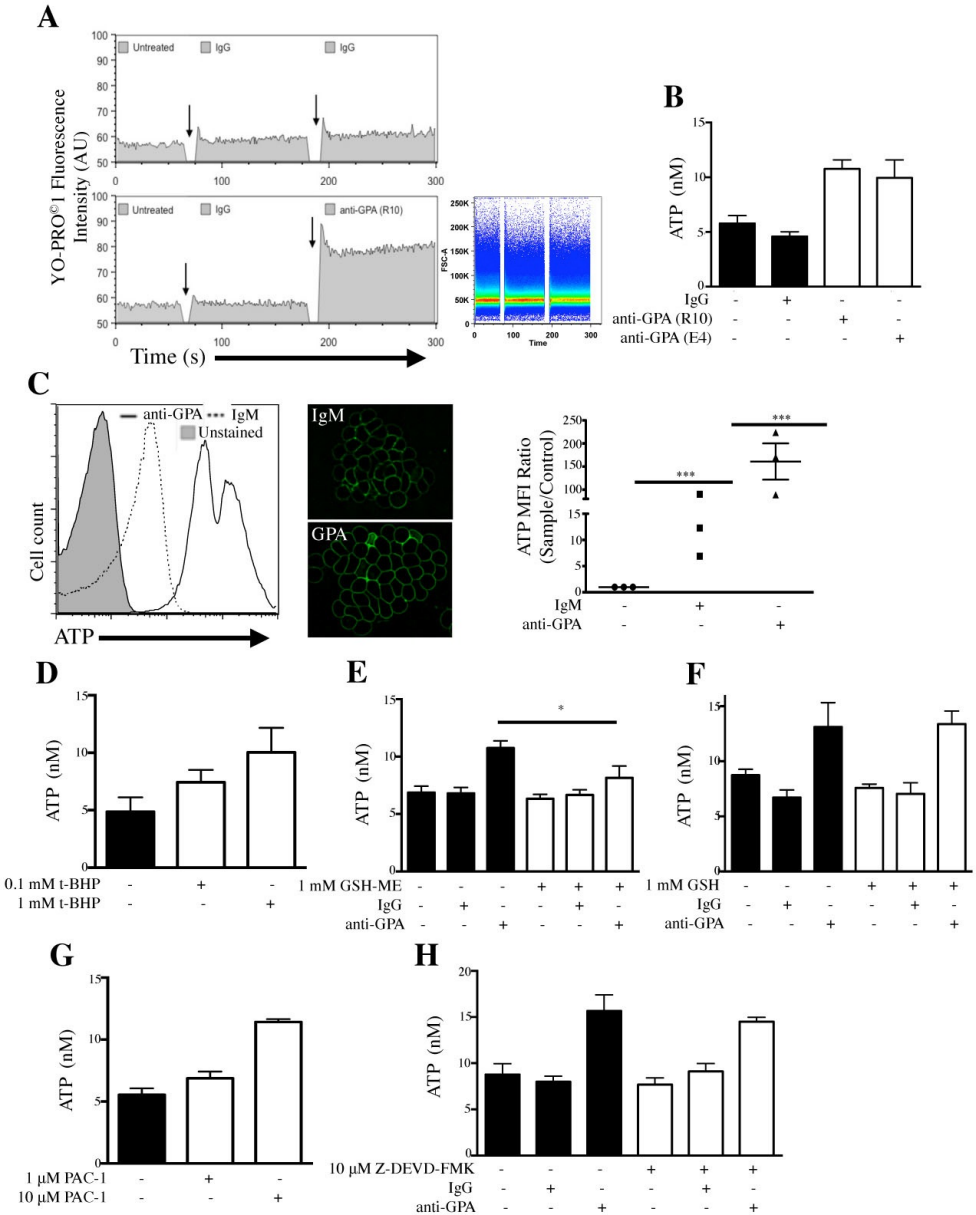
GPA-ligation opens a channel, promotes ATP release through ROS. (A) GPA ligation permeabilizes RBCs for YO-PRO^®^-1 iodide (YO-PRO). Cells were incubated with YO-PRO and analyzed by kinetics using flow cytometry. Baseline values were acquired followed by addition of IgG control Ab and then either IgG control (top) or anti-GPA mAb (R10) (bottom). Arrows indicate time point of antibody addition. The size distribution of RBCs is also shown (right panel). (B) Ligation of GPA mAbs induces ATP release. RBCs were incubated with IgG, anti-GPA mAb (R10 and E4) and ATP release was measured. (C) GPA-mediated ATP release measured by ATP-specific fluorescent probe, 2-2Zn(II). RBCs were pretreated with 1 μM 2-2Zn(II) prior to incubation with either control IgM or anti-GPA **mAb**. ATP release was measured by flow cytometry. RBCs were imaged by wide-field fluorescence microscopy followed by deconvolution. (D) ROS generation in RBCs induces ATP release. RBCs treated with buffer (black bar) or two concentrations of t-BHP. (E) Cell permeable GSH-ME reduces GPA-mediated ATP release. RBCs were incubated with buffer (black bars) or 1 mM GSH-ME. RBCs were then treated with anti-GPA mAb (R10) or IgG control mAb, and ATP measurements were acquired. (F) Extracellular ROS does not affect GPA-mediated ATP release. RBCs were incubated with buffer or 1 mM non-cell permeable glutathione (GSH) and then treated with anti-GPA mAb (R10) or IgG control mAb. (G) Caspase-3 activator PAC 1 induces ATP release in RBC. RBCs were pre-incubated with 1% DMSO (control) or two concentrations (1 and 10 μM) of PAC 1 before ATP measurements were determined. (H) Inhibition of caspase-3 does not reduce GPA-mediated ATP release. RBCs were pre-incubated (RT x 10 min) with 0.1% DMSO (control) or the cell-permeable caspase-3 inhibitor Z-DEVD-FMK (10 μM). RBCs were treated with anti-GPA mAb (R10) or IgG control mAb (both 5 μg/mL).

To determine if a pore large enough to allow ATP (375 Da) release opens following GPA ligation, we incubated RBCs with YO-PRO^®^-1 iodide, a cell impermeant nucleic acid stain that has been used to quantify gap junction channels, a family of pores that allow passage of small molecules such as ATP [29]. Ligation of GPA using the R10 epitope **(Fig 3A, bottom panel)** induced an increase in RBC-associated fluorescence from 58 to 78 AU when compared to control Ab (top panel). To ensure that this increase was not the result of antibody-induced cell aggregation, we measured the size distribution of RBCs before and after GPA ligation (**Fig 3A, right panel)**. Having determined that a membrane pore was opening following GPA ligation, we investigated whether ATP was simultaneously released. RBCs incubated with anti-GPA (R10 and E4) significantly increased ATP concentration in the supernatant from 4.6 ± 0.4 nM to 10.8 ± 0.8 nM (R10) and 10.0 ± 1.6 nM (E4) (*p* < 0.008) (**Fig 3B**). We next incubated RBCs with a fluorescent ATP probe 2–2 Zn(II), which binds to the extracellular side of the plasma membrane and fluoresces upon interaction with ATP as it is released. [19] GPA ligated RBCs showed a significant increase in ATP release compared to control, from MFI = 39.6 to MFI = 1282 (unstained MFI = 5.3) (**Fig 3C, left panel)**. This dye promotes cell aggregation due to its lipophilic nature [19], however this did not prevent observing fluorescent ATP levels for each cell. A similar increase in fluorescence intensity was observed using wide field fluorescence 3-D acquisition followed by deconvolution (**right panels)**. RBCs incubated with t-BHP (100 μM and 1 mM) also released increased amounts of ATP, from 5.4 ± 1.2 nM to 7.4 ± 1.1 nM and 10.0 ± 2.1 nM, respectively (*p* < 0.01) (**Fig 3D**). Pretreatment of RBCs using the cell-permeable glutathione monoethyl ester (GSH-ME, 1 mM) significantly decreased the amount of ATP following GPA ligation, from 10.8 ± 0.6 nM to 8.2 ± 1.0 nM (*p* < 0.008) (**Fig 3E**). However, pretreatment under similar conditions with cell impermeable glutathione (GSH) had no effect (anti-GPA = 13.1 ± 2.2 nM, anti-GPA + GSH = 13.4 ± 1.2 nM, *p* = 1) (**Fig 3F**), suggesting that the extracellular ROS-based autocrine effect on RBCs was not required for the GPA-mediated ATP release. Next, we asked if caspase-3 played a role in ATP release following GPA-ligation. Incubation of RBCs with caspase-3 activator PAC 1 (1 and 10 μM) triggered a significant increase in ATP release, from 5.5 ± 0.5 nM to 6.9 ± 0.5 nM and 11.4 ± 0.2 nM, respectively (*p* < 0.02) **(Fig 3G)**. However, pre-incubation of RBCs with a caspase-3 inhibitor, FAM-DEVD-X, prior to GPA ligation did not significantly alter ATP release (*p* = 0.2) **(Fig 3H**). Our data suggest that GPA ligation-induced ATP release is dependent on intracellular but not extracellular ROS levels, and although GPA-mediated ROS production activates caspase-3, this signaling may not be directly linked to GPA and ROS-induced ATP release.

Several pores and channels have been described to be functionally associated with ATP release in RBCs such as pannexins, MRPs, and CFTR [30–32]. In particular, CFTR expression and its involvement with ATP release was previously shown in murine RBCs [31]. Despite confirming the presence of CFTR on human RBCs by Western blot (S2 Fig), we were unable to confirm its role in GPA-mediated ATP release using the specific inhibitor GlyH-101. Similarly, MRP channels and purinergic receptors were not found to be independent conductors for ATP released from RBCs secondary to GPA ligation (S2 Fig).

### Intracellular ROS production decreases membrane deformability

**Fig 4:**
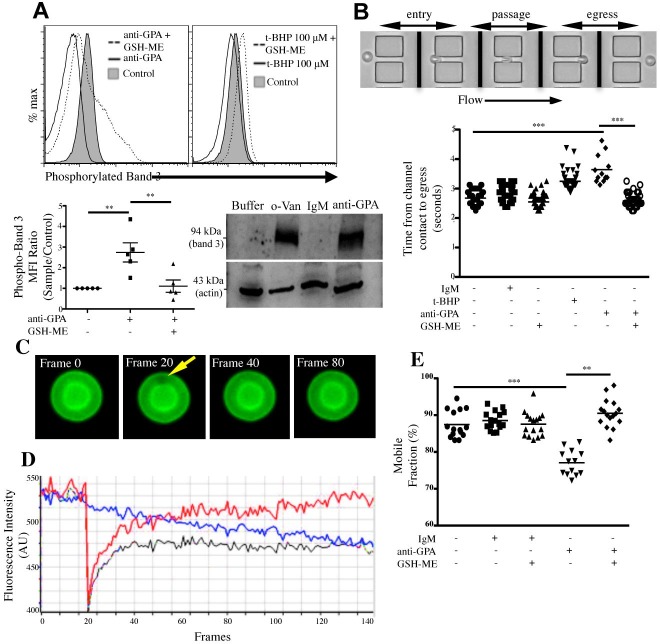
RBC ROS production induces Band 3 phosphorylation, decreasing membrane deformability and lipid mobility. (A) GPA mediated ROS production increases band 3 phosphorylation. RBCs pretreated with buffer or 1 mM GSH-ME in the presence of EMA were then incubated with anti-GPA mAb (E4, 1 μg/mL or R10, 5 μg/mL), IgG control mAb (5 μg/mL, all top left panel), or t-BHP (100 and 200 μM, top right panel). RBCs were washed twice and analyzed by flow cytometry. Bottom right panel: Western blot of RBCs treated with IgG control, anti-GPA, or Na_3_VO_4_. Samples were probed with anti-phosphotyrosine. (B) GPA-mediated ROS production decreases membrane deformability. RBCs were incubated with either control or anti-GPA (E4) Ab (1ug/ml) in the presence or absence of GSH-ME (1 mM). Sequential images (3 frames/sec) were recorded of each cell’s passage through the channel. (C) Serial snapshots of a RBC during a standard FRAP experiment, with a 1 x 1 μm targeted area for bleaching. (D) Standard FRAP recovery curve. Black line: the original recovery curve measured in the bleached area, red line: the photobleaching rate measured on the same cell away from the bleached area, blue curve: the final recovery rate calculated using Slidebook. (E) Treatment of RBC with GPA decreases lipid recovery rate, which is partially restored by GSH-ME. RBCs were pretreated with 1μM GSH-ME for (RT x 5 min) and then incubated with control or anti-GPA antibody (1 μg/ml, RT x 10 min).

We have previously reported that during chronic inflammations such as in SLE and trauma, RBCs undergo alteration in the phosphorylation levels of β-spectrin and band 3 [5,6]. Importantly, band 3 phosphorylation was recently reported to depend critically on protein kinase p72 Syk, an enzyme that is activated by increased intra-RBC ROS [33]. We next investigated the effect of RBC GPA ligation on tyrosine phosphorylation of band 3, and its dependence on ROS. We used eosin-5-maleimide (EMA) as an indirect marker for band 3 phosphorylation. The efficiency of EMA binding to lysine 430 on the extracellular loop of band 3 was shown to parallel the levels of band 3 tyrosine phosphorylation, although the details of the binding are currently poorly understood [34]. Our data show that anti-GPA mAb (E4) induced band 3 phosphorylation (E4 MFI = 25.5, control MFI = 14.6) (**Fig 4A, top left panel**), an increase that was significantly reduced when RBCs were pretreated with GSH-ME (E4 MFI = 14.9). Also, incubation of RBCs with 100 μM t-BHP triggered an increase in band 3 phosphorylation from MFI = 14.6 to 17.7 (**top right panel)**. In both experiments, band 3 phosphorylation was reduced following pretreatment with GSH-ME (100 μM t-BHP MFI = 6.4), suggesting that the baseline phosphorylation levels of band 3 depend partially on ROS. This was further confirmed by Western blotting **(bottom right panel)** using anti-phosphotyrosine, pY20 (Biosource) as a developing antibody. As a positive control, RBCs were incubated with sodium ortho-vanadate. We next asked if the effect of GPA ligation on membrane deformability is a result of excessive ROS generation, and if ROS scavengers can prevent it. We used 2-D microchannels to assess RBC deformability, measured as the time required for RBCs to pass through a capillary-like array (**Fig 4B, top panel**). Ligation of GPA increased the average time required for RBCs to pass through the capillary-like channel, from 2.7 ± 0.2 s to 3.7 ± 0.5 s (*p* < 0.01). However, GPA-ligated RBCs in the presence of GSH-ME required less time to deform and move through the channels (2.6 ± 0.2 s, *p* < 0.001), suggesting that intracellular ROS is in part responsible for the GPA-mediated decrease in membrane deformability (**Fig 4B, bottom panel)**.

### GPA-mediated ROS production inhibits lipid mobility

We next investigated the role of GPA-mediated ROS production on plasma membrane lipid mobility using fluorescence recovery after photobleaching (FRAP). **Fig 4C** shows serial images of a representative RBC during a FRAP experiment, while a corresponding recovery rate of the membrane at the specified region is shown in **Fig 4D**. GPA-ligated RBCs resulted in a significant reduction in the mobile fraction from 87.5 ± 3.7% in control IgM-treated RBCs down to 77.4 ± 3.4% (*p* < 0.0001) (**Fig 4E**). Our results also show that pretreatment with GSH-ME restored the GPA-mediated loss in lipid mobility, with a mobile fraction of 91.0 ± 4.0% compared to 77.4 ± 3.4% (*p* < 0.0001).

### Complement-altered RBCs from septic patients partially recapitulate the functional phenotype of GPA-ligated RBCs

**Fig 5:**
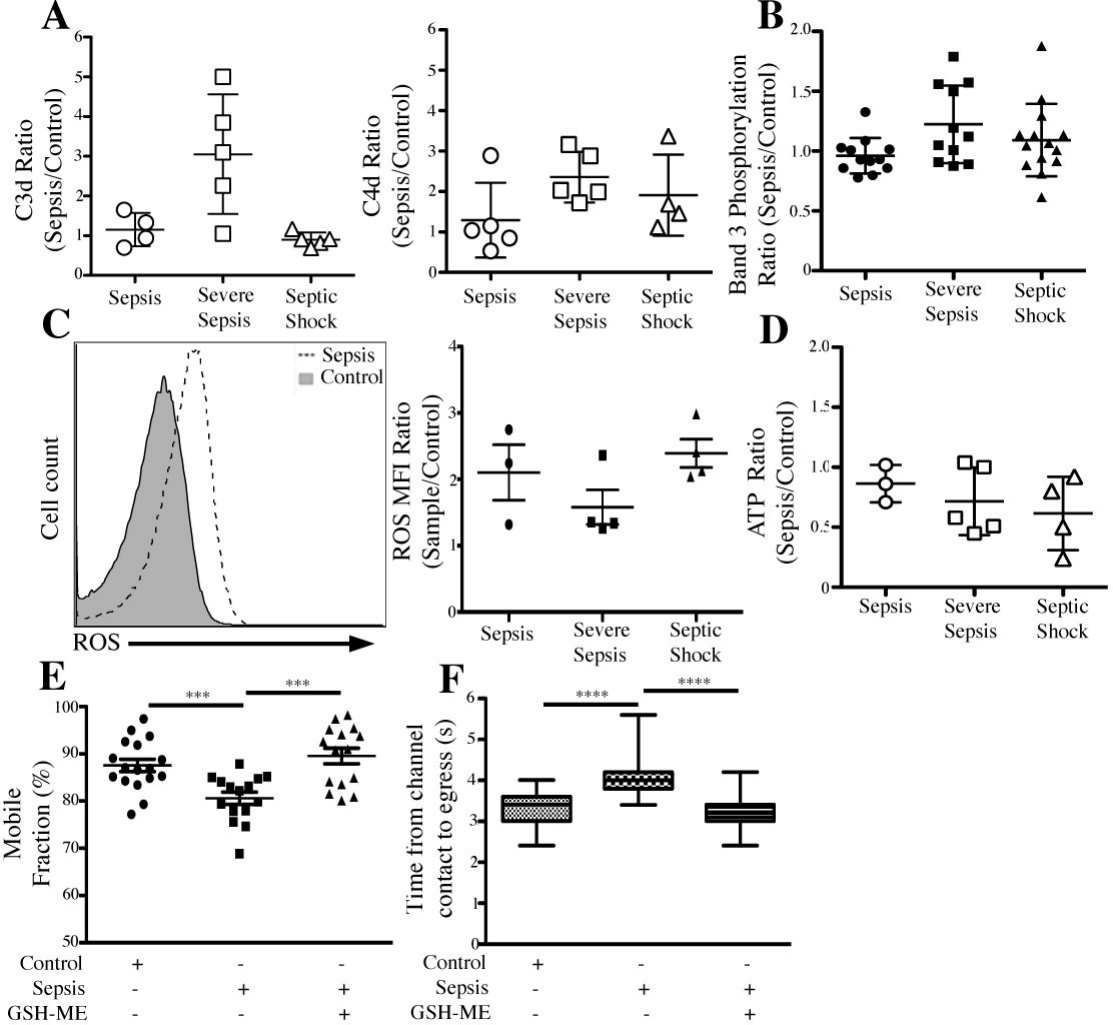
RBCs from Septic Patients partially recapitulates functional phenotype of GPA-ligated RBCs. (A) RBCs from septic patients show increased presence of C4d fragments, but not C3d. RBCs isolated from patient samples were incubated with anti-C3d or anti-C4d and analyzed by flow cytometry. (B) RBCs from septic patients do not show uniform increase in band 3 phosphorylation. RBCs from septic and control patients were incubated with EMA, washed twice, and band 3 phosphorylation was measured by flow cytometry. (C) ROS levels increased in septic RBCs. RBCs were incubated with DHR 123, washed twice, and measured for intracellular ROS by flow cytometry. (D) RBCs from septic patients release less ATP than control RBCs. RBCs from septic and control patients were isolated and ATP concentration was measured as described in Methods. (E) Sepsis-induced decrease in RBC lipid mobility depends on ROS. RBCs from control and septic patients were incubated with DiO lipid dye in the presence or absence of GSH-ME (1 mM) prior to running FRAP. (F) Sepsis-dependent loss of RBC membrane deformability depends on ROS. RBCs from control and septic patients were washed and loaded into a 2D microfluidic device in the presence or absence of GSH-ME (1 mM, RT x 15 min). Sequential images (3 frames/sec) were recorded of each cell’s passage through the channel.

RBCs from 27 sepsis patients and 22 controls were analyzed for complement deposition, ROS production, band 3 phosphorylation, ATP release, membrane deformability, and lipid mobility. Not all patient samples were investigated for all functional parameters. The results are presented as ratios between the measured value of septic over control RBCs. **Fig 5A** shows that in the case of C4d fragments (right panel), deposition of RBCs from septic patients was more pronounced in severe septic and septic shock when compared to controls, whereas C3d fragments in (left panel) septic shock patients had levels not different than uninfected controls. RBC band 3 phosphorylation was not uniformly increased in sepsis patients; only a few patients displayed increased levels of band 3 phosphorylation, without a clear correlation between the RBC complement levels and the intensity of band 3 staining **(Fig 5B**). Conversely, intra-RBC ROS concentrations were increased in all septic samples studied (**Fig 5C)**. When measuring the ATP concentration in the supernatant, we observed a gradual decrease in the amount of ATP that correlated inversely with the severity of sepsis (**Fig 5D**). Consistent with our *in vitro* FRAP data RBC lipid mobility of 4 patients with septic shock (**Fig 5E)** was significantly decreased (Mf_control_ = 87.5%, Mf _sepsis_ = 80.6%, *p* < 0.0008), when compared to matched controls. Consistent with previous reports [35–37], RBCs from septic shock patients also showed a significant decrease in membrane deformability (**Fig 5F)**, as measured by 2-D microchannel arrays (t_control_ = 3.2 seconds, t_sepsis_ = 4 seconds, *p* < 0.0001). Moreover, RBCs isolated from septic patients treated with 1 mM GSH-ME for 15 minutes prior to functional analyses partially restored both membrane deformability (from 4 to 3.2 seconds) as well as lipid mobility (from Mf_control_ = 80.6% to Mf_sepsis_ = 89.6%), suggesting that the beneficial microcirculatory effects of antioxidant therapy reported in sepsis [38] could also be attributed to its effect on the mechanical properties of circulating RBCs.

## Discussion

In this study, we investigated the deleterious effects of RBC ROS production promoted by GPA ligation using both *in vitro* and *ex vivo* approaches. Our data demonstrate that ligation of GPA by antibody initiates a NOX-dependent increase in ROS production. In turn, elevated intracellular ROS triggers multiple signaling cascades culminating in loss of RBC mechanical functions and ATP release, both of which could partially explain the flow alterations described in sepsis. Others have shown that Rac GTP-ases and PKC contribute to greater oxidative stress in sickle cell RBCs by increasing erythrocyte NOX activity [27]. Our data show a similar signaling pathway whereby GPA ligation activates Rac 1, increasing NOX activity, and generating ROS. However, the GPA-mediated ROS production seems not to fall under the classical NOX activation pathway involving activation of PKC and phospholipase A2 [39]. Both steps require Ca^++^, yet engagement of GPA by antibody does not promote a measurable Ca^++^ influx. Furthermore, inhibition of PKC with calphostin C prior to GPA ligation does not prevent ROS production, suggesting that additional mechanisms involved in activation of NOX play a role in GPA-mediated ATP release.

Human RBCs contain large amounts of procaspase-3, considered a leftover erythropoiesis protein [40]. In nucleated cells, reports show a correlation between increased intracellular ROS and programmed cell death by direct activation of caspase-3 [28]. Our results show that GPA activates caspase-3, but in a different manner than t-BHP; whereas t-BHP generated cell-wide activation, resulting in uniform staining, GPA-ligation activated caspase-3 in a punctuated, well-defined pattern. Localized, transient, functional domains have been described in RBCs as a result of specific signaling pathway responses [41]. It is conceivable a similar mechanism is responsible for limited activation of caspase-3, although they did not co-localize with GPA molecules.

Recently, a novel role for ATP in RBC mechanical properties has been uncovered [42–47]. We here have showed using fluorescence microscopy and flow cytometry using a novel ATP probe [19] that GPA ligation by antibody induces a robust increase in ATP released by RBCs. This increase was significantly reduced in the presence of additional antioxidants such as the cell-permeable GSH-ME. While we observed a significant ATP release in RBCs treated with PAC-1, further experiments did not support our hypothesis that this release was causally linked to GPA ligation **(Fig 2G and 2H)**. It is therefore likely that increased ROS levels induce multiple responses from RBCs including caspase-3 activation and ATP release.

We previously reported an inability to identify pannexin-1 in human RBCs using flow cytometry or immuno-blotting methods [19]. However, MRP1 and MRP4 were previously involved in ATP release, and both were identified in human RBCs by mass spectrometry [48]. Using the specific inhibitor MK-571, we showed RBC MRP1 is involved in CR1-mediated ATP release [19]. Moreover, previous reports show mice with CF having increased ATP concentrations in blood [31], while others have reported the importance of purinergic receptors as regulators of ATP release in osteoclasts [49]. In spite of promising data, we could not confirm the role of any of these channels in mediating ATP release following GPA ligation.

Our data obtained using RBCs isolated from septic patients paradoxically showed an opposite trend from our *in vitro* results. Even though there is an increased activity of plasma ecto-nucleases in sepsis that directly lowers plasma ATP concentrations due to bacterial presence and generalized inflammation [50,51], our experiments using isolated and purified RBCs suggest that another mechanism is responsible for diminished RBC ATP release in sepsis. Increased concentrations of extracellular ADP reversibly inhibit RBC ATP release by acting on P2Y13 receptors on RBC membranes through a c-AMP dependent mechanism [52]. During septic episodes, the blood concentration of ADP increases 6–8 times over control (from 1.2 to 8.4 μM/L), decreasing to near normal values 72 hours post-sepsis [53]. Patients in our study were enrolled within a 24-hour window post admission, providing a possible explanation for the observed, *ex vivo* low ATP release by complement-altered RBCs isolated from septic patients.

Our results regarding C3d levels on circulating RBCs from sepsis patients were indistinguishable from control samples. In humans, C3 fragments present on RBC membranes have been shown to decrease RBC membrane deformability [7] and significantly shorten the half-life of circulating RBCs compared to RBC-bound C4 fragments [54]. It is possible that, at the onset of the disease, complement is not fully activated, whereas after time, the less deformable RBCs are trapped in the spleen and liver or removed from circulation. This mechanism could provide an explanation as to why C4 fragments are present on RBC membranes from sepsis patients, while the C3b-RBCs are missing.

Septic patients display a wide array of microcirculatory flow disturbances [55–58]. Consistent with our *in vitro* data, our *ex vivo* functional results indicate that RBCs from septic patients show a significant decrease in RBC membrane deformability and lipid mobility. These results indicate that the marked changes in membrane deformability due to GPA ligation either by complement fragments C3b, C4b, or antibodies are the result of GPA-specific signaling cascades rather than passive, antibody-driven cross-linking of membrane proteins.

In summary, our findings regarding GPA engagement by complement fragments or autoantibodies reveal a novel and detrimental role of circulating RBCs during inflammatory conditions. Overwhelming intracellular ROS concentrations ultimately impair critical RBC mechanical capabilities, thus disrupting their movement and interactions within the microvasculature. The data point to complement-altered RBCs as possible effector cells, actively influencing nearby cells through release of bioactive molecules such as ROS and ATP.

## Supporting Information

S1 Fig t-BHP produces ROS in RBCs. Treatment of RBCs with tert-butyl hydroperoxide (t-BHP) induces ROS. RBCs were incubated with 10 or 100 μM t-BHP in the presence of 5 μM DHR 123. Cells were washed twice and recorded by flow cytometry. The left panel is a representative experiment and the right panel is the cumulative data taken from all experiments.
(JPG)

S2 Fig MRP and CFTR channels not solely responsible for GPA-mediated ATP release. (A) Detection of CFTR by immunoblotting. Ghosted RBC membranes (non-boiled) were resuspended in 1x LDS Sample Buffer, separated using SDS NuPAGE, and transferred onto nitrocellulose membrane. Mouse lung lysate served as positive control (lane 3); lane 2 was left empty. (B) CFTR Inhibitor raises baseline ATP levels in RBC. RBCs were pre-incubated with DMSO (control) or two concentrations of GlyH-101. ATP concentration was determined as described in Experimental Procedures. (C) Inhibition of CFTR does not reduce GPA-mediated ATP release. RBCs were pre-incubated with 0.1% DMSO (control) or 1 μM GlyH-101. RBCs were then washed twice with buffer containing1 μM GlyH-101 and ligated with anti-GPA mAb (R10) or IgG control mAb (5 μg/mL). Extracellular ATP concentration was determined as described in Experimental Procedures. (D) Indomethacin does not inhibit GPA-mediated ATP release. RBCs were pre-incubated with 0.1% DMSO (control) or 10 μM indomethacin. RBCs were then ligated with either control IgG or anti-GPA mAb. ATP concentration was determined as described in Experimental Procedures. (E) MRP channels are not responsible for GPA mediated ATP release. RBCs were pre-incubated with buffer or 10 μM MK-571, a known specific of MRPs. RBCs were then processed as outlined in methods with the antibodies given during the last resuspension step. ATP concentration was determined as described in Experimental Procedures. (F) Inhibition of P2 receptors does not reduce GPA-mediated ATP release. RBCs were pre-incubated (RT x 10 min) with buffer or either 10 μM PPADS (left graph), *iso*-PPADS (middle graph) or suramin (right graph). RBCs were then processed as outlined in methods with the antibodies given during the last resuspension step. ATP concentration was determined as described in Experimental Procedures.
(JPG)
