# Supplementary figures and images for: Ligation of Glycophorin A Generates Reactive Oxygen Species Leading to Decreased Red Blood Cell Function
Source: PLoS One. 2016 Jan 19;11(1):e0141206. doi: 10.1371/journal.pone.0141206 (PMC4718526; doi:10.1371/journal.pone.0141206)

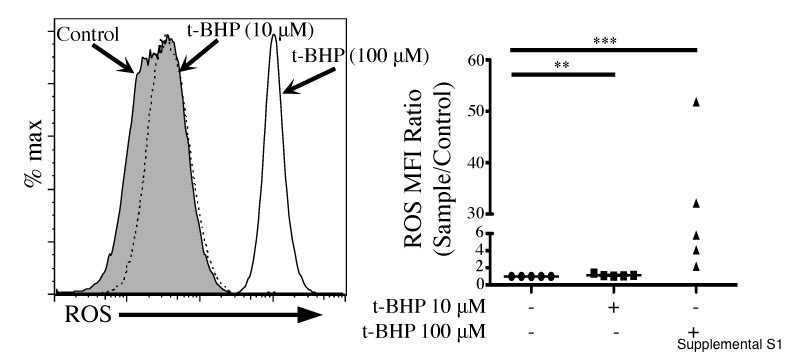

Supplement: S1 Fig — Treatment of RBCs with tert-butyl hydroperoxide (t-BHP) induces ROS. RBCs were incubated with 10 or 100 μM t-BHP in the presence of 5 μM DHR 123. Cells were washed twice and recorded by flow cytometry. The left panel is a representative experiment and the right panel is the cumulative data taken from all experiments. (JPG) [file pone.0141206.s001.jpg]

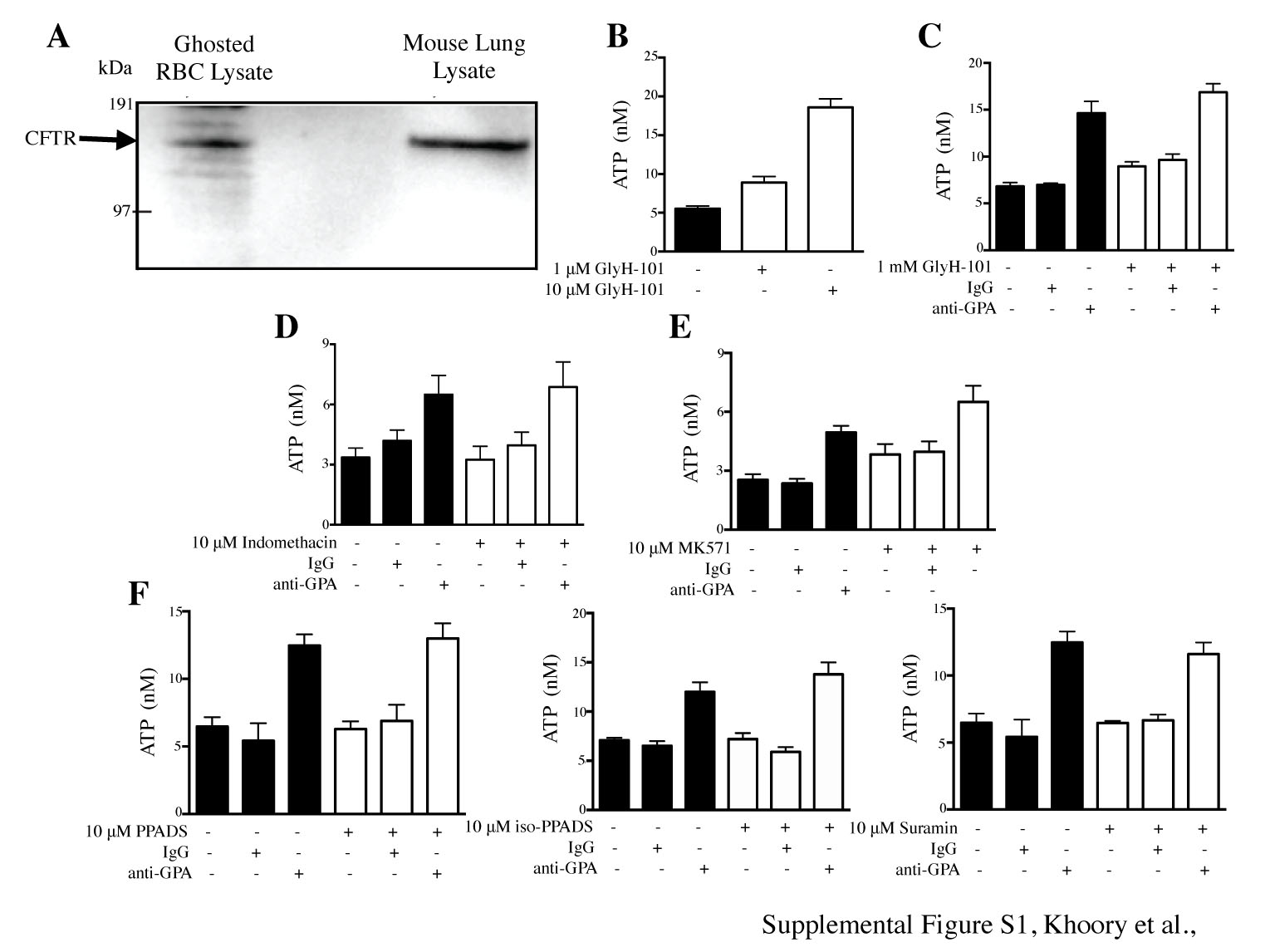

Supplement: S2 Fig — (A) Detection of CFTR by immunoblotting. Ghosted RBC membranes (non-boiled) were resuspended in 1x LDS Sample Buffer, separated using SDS NuPAGE, and transferred onto nitrocellulose membrane. Mouse lung lysate served as positive control (lane 3); lane 2 was left empty. (B) CFTR Inhibitor raises baseline ATP levels in RBC. RBCs were pre-incubated with DMSO (control) or two concentrations of GlyH-101. ATP concentration was determined as described in Experimental Procedures. (C) Inhibition of CFTR does not reduce GPA-mediated ATP release. RBCs were pre-incubated with 0.1% DMSO (control) or 1 μM GlyH-101. RBCs were then washed twice with buffer containing1 μM GlyH-101 and ligated with anti-GPA mAb (R10) or IgG control mAb (5 μg/mL). Extracellular ATP concentration was determined as described in Experimental Procedures. (D) Indomethacin does not inhibit GPA-mediated ATP release. RBCs were pre-incubated with 0.1% DMSO (control) or 10 μM indomethacin. RBCs were then ligated with either control IgG or anti-GPA mAb. ATP concentration was determined as described in Experimental Procedures. (E) MRP channels are not responsible for GPA mediated ATP release. RBCs were pre-incubated with buffer or 10 μM MK-571, a known specific of MRPs. RBCs were then processed as outlined in methods with the antibodies given during the last resuspension step. ATP concentration was determined as described in Experimental Procedures. (F) Inhibition of P2 receptors does not reduce GPA-mediated ATP release. RBCs were pre-incubated (RT x 10 min) with buffer or either 10 μM PPADS (left graph), iso-PPADS (middle graph) or suramin (right graph). RBCs were then processed as outlined in methods with the antibodies given during the last resuspension step. ATP concentration was determined as described in Experimental Procedures. (JPG) [file pone.0141206.s002.jpg]
